# Oral Reports in an Organic Chemistry Laboratory Curriculum

**DOI:** 10.1021/acs.jchemed.5c00889

**Published:** 2026-06-23

**Authors:** Sara A. Mehltretter, Neil Schmitzer-Torbert, Laura M. Wysocki

**Affiliations:** † Department of Rhetoric, 2719 Wabash College, 301 W. Wabash Ave., Crawfordsville, Indiana 47933, United States; ‡ Department of Psychology, Wabash College, 301 W. Wabash Ave., Crawfordsville, Indiana 47933, United States; § Department of Chemistry, Wabash College, 301 W. Wabash Ave., Crawfordsville, Indiana 47933, United States

**Keywords:** second-year undergraduate, laboratory instruction, organic chemistry, communication, writing, testing, assessment, aldehydes, ketones, esters, NMR spectroscopy

## Abstract

The importance of teaching chemistry undergraduate students communication skills is widely recognized, but laboratory assessment is dominated by written reports with delayed feedback from instructors. The literature has demonstrated encouraging learning outcomes like positive student attitudes and deeper understanding associated with oral assessments, alongside the significant benefits of immediate feedback, but logistical challenges remain regarding implementation and consistent assessment across different instructors. This report describes an oral lab report in Organic Chemistry II that utilizes a technical, conversational approach and addresses some of the challenges associated with oral assessments, while retaining their previously documented benefits, including the development of oral communication skills. Using a rubric that is focused on principles of the Science Writing Heuristic, a comparison of students’ overall score on written and oral lab reports showed that students scored higher on the oral lab report than with an analogous rubric on the written lab reports for a given experiment. Additionally, students score significantly higher on their oral report when compared to their written reports for other experiments that have a similar complexity. Oral reports can be conducted in-person or virtually without a significant difference in scoring. The challenge of instructor bias in scoring is discussed and instructor reflections are included.

## Graphical abstract

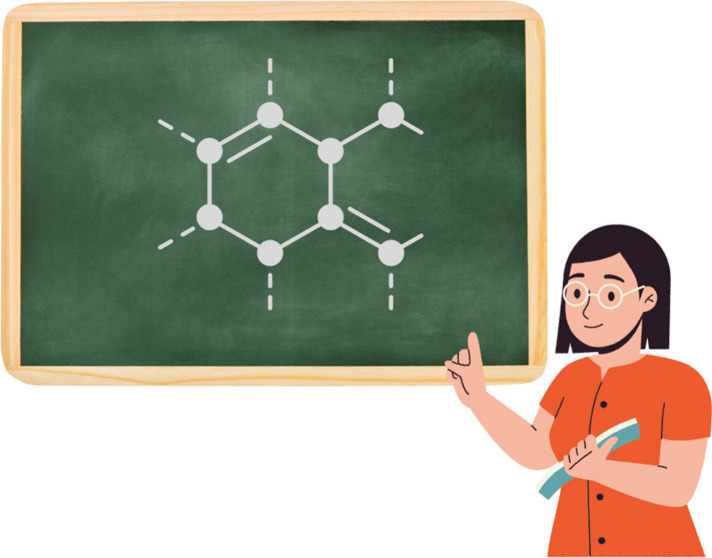

## Introduction

Communication is important for scientists to learn^1^ and professional chemists communicate their work to build collaborations, prepare grant proposals, report results, and more. Communication categories and styles vary,^2^ including oral,^3^ written,^4^ and/or visual;^5^ formal or informal; technical or translational; and for different audiences:^6^ scientists with related or unrelated expertise, public, or media;^7^ collaborator, evaluator, or learner. Allowing students to practice a variety of communication strategies prepares them for future endeavors. However, students in laboratory courses are most often asked to communicate their results in written lab reports.

Historically, oral assessments were common,^8^ and they have numerous benefits. They provide an opportunity to practice oral communication that can translate^9^ to situations students encounter in their professional lives. Oral assessment encourages exchanges and refinement of ideas that often lead to deeper understanding^10–13^ and may be more inclusive for students with disabilities.^14,15^ They also probe student learning without access to AI-generated responses, a growing concern in writing assignments, including lab reports.^16–18^

A key factor in student development is effective, timely feedback.^19^ Giving high-quality, written feedback is time-intensive for instructors. In written lab reports, feedback is delayed and students may not read it or understand the message behind written phrases.^4,20,21^ Many oral assessments feature immediate, verbal feedback with an opportunity for students to ask clarifying questions.

On the other hand, oral assessments have several drawbacks, many of which are logistical. With large-enrollment classes, scheduling oral assessments becomes an unreasonable task.^10,13,19^ A greater number of lab sections and grading responsibilities for teaching assistants generate concerns about grader bias.^20,21^ Written assessments are easier to compare, ensuring grading consistency. Students are more familiar with written assessments, and a novel oral interview may introduce stress that could negatively affect performance.^10,12,13,19,22^

Oral assessments come in many forms, and recent examples from the chemistry community show the diversity and ingenuity of pedagogical interventions, including a content-based exam,^10,13,22^ group oral assignment,^23^ video recording of a laboratory experiment,^24^ or brief reflective interview to complement a written lab report.^11^ In all of these cases, there is evidence for benefits in student learning and student attitudes.^25^ A recent phenomenology study of students’ perceptions of regularly conducted oral assessments for a biochemistry laboratory course highlighted themes surrounding the interview’s ability to reveal gaps in knowledge: the structure encouraged deeper understanding, students experienced uneasy feelings, and they prepared and carried out the experiment differently.^12^ While feelings of unease can have positive outcomes like greater preparation, they can also lead to negative outcomes in student performance. These authors identified that challenges remain when implementing oral assessments in introductory or large-enrollment courses. The assessment we describe seeks to address challenges related to implementation and provide an easier incorporation path for oral lab reports, including a single exposure activity with a familiar format and the possibility of multiple instructors administering the assessment, while maintaining positive learning outcomes traditionally associated with oral assessments.

## Methods

### Institution and Course Background

The oral lab report was implemented at a small, private, residential liberal arts college for men. Organic Chemistry II includes three 50 min lectures and one 3 h lab per week, which students complete in the same semester. Enrolled students are usually chemistry or biochemistry majors, chemistry minors, or pursuing healthcare careers. While the corresponding author (L.M.W.) is the course’s lead instructor, the COVID-19 pandemic necessitated an additional lab section taught by a second instructor, who also administered the oral lab report. This research study was approved by the Wabash College Institutional Review Board (#1410201). All participants provided informed consent by typing their name in a digital consent form (spring 2021) or signing a paper consent form (spring 2022) for their class performance and survey responses to be included in the analyses presented here. All data were deidentified prior to analysis. No unexpected or unusually high safety hazards were encountered during this activity. Though this study focuses on spring 2021 and 2022, instructors continue to use this activity each spring.

Several similarities exist between this work and previous phenomenological research on oral assessments in biochemistry.^12^ Both courses have a flipped prelab format, a low-stakes prelab quiz, and a question-and-answer session to help students prepare for the experiment. Students are encouraged to take notes during the experiment, which they can use during the oral assessment that is scheduled within the following week, but the written notes are not graded. In the study described here, the interviews are shorter (15 min vs 30 min), students only complete one oral assessment with the rest of the experiments assessed by a written report, and Organic Chemistry II is typically found earlier in the curriculum than Biochemistry.

### Science Writing Heuristic

To set clear expectations for students and decrease stress related to oral performance, we sought a consistent organization for the written and oral lab reports. We were inspired by the Science Writing Heuristic (SWH), a tool for learning that has been used at different instruction levels as a way to encourage students to generate meaning from their experience and data.^26–30^ Though we modified some of the components of the SWH, students regularly used this framework in their weekly lab reports in the prerequisite course, Organic Chemistry I.

Reports in the literature point to anxiety and stress about the unknown experience of oral assessment as a potential drawback.^10,12,13,22,23^ By making the format of the oral report parallel to the familiar written report, students had a better idea of where to begin their preparation. We also generated an analogous grading rubric for the written and oral lab report for each experiment (see the Supporting Information).

### Oral Interview

The time necessary to administer oral assessments is often discussed as a limitation, especially for introductory and large courses.^10,13,22^ Here, several measures allowed for reasonable scheduling. Three experiments were identified that were worth the same number of points, occurred in the first half of the semester, had similar levels of complexity in analysis, and focused on carbonyl chemistry. One dealt with a Wittig olefination, another involved acetal formation,^31^ and a third used Fischer esterification.^32^ For these experiments, the instructor had one-third of the class giving oral reports and two-thirds of the class assigned written reports. In a class of 30–45 students, this left 10–15 appointments in a week’s time.

Most interviews were done in-person, but COVID-19 necessitated virtual interviews; both used a consistent format. Each experiment had a script of questions (Supporting Information), but some threads tied them together. The instructor set the tone for a conversational assessment that would sound like the familiar lab report format but may include follow-up questions and the chance for students to describe their thought process and self-correct if appropriate. While the instructor used the script for each experiment, there was freedom to follow up with more specific questions, especially when students needed guidance to gain a better understanding. In general, these appointments stayed within 15 min, unless the student initiated a more in-depth discussion. This assessment allowed for several accommodations that could be helpful for students with disabilities or non-native speakers:^14,15^ the format is flexible (virtual or in-person), core questions are similar to written report requirements, students are allowed to bring prepared notes, the time could be extended, and questions may be rephrased for clarity.

### Assessment

We developed a common rubric for all oral lab reports, regardless of the experiment. While the categories are general to accommodate different experiments, the oral report rubric is analogous to the written report rubric for these experiments, with an emphasis on SWH components. Some points were reallocated where appropriate to accommodate the different ways learning is demonstrated in written and oral formats. For a direct comparison, all rubrics are available in the Supporting Information.

The instructor asked guiding questions if a student responded incorrectly to a question, but there was a grade penalty if the student did not self-correct. Thereby, students got verbal feedback on their ideas in real-time, but their score reflected performance. The instructor wrote comments on the rubric and scoring was completed immediately after the oral interview. Students received a copy of this rubric within an hour of their lab report.

## Results

### Comparison of Oral and Written Report Grade Outcomes

To measure the success of the oral lab reports, we first compared scores for oral reports to those for written ones. This approach is limited by differences in the ways that students demonstrate learning in oral and written formats. Thus, a direct comparison of scores cannot determine if one format is superior to the other in terms of achieving the learning objectives for the experiment. However, a comparison of scores on both formats is useful because of its importance for student attitudes and beliefs about the accuracy of course grades, especially for an oral assessment that only occurs once. Even if both oral and written lab reports were equally effective in achieving lab objectives, if students were to receive lower scores for an oral report, which is a less common format for our institutional context, it could negatively affect student attitudes and experience in the course.

While the distribution of oral lab reports across three experiments was a solution to scheduling challenges, it allowed a comparison of the oral and written report scores within the same experiment. A total of 52 students completed these assessments in two offerings of the Organic Chemistry II lab, which were assessed using the rubrics available in the . In all three experiments, students scored significantly higher in the oral report compared to the written report (see the Supporting Information for statistical comparison).

Another way to look at the data is to consider the difference in scores for an individual on their oral report compared to the average of the same student’s written reports for the other two experiments in this set of three. Again, the oral report compares favorably to the written report, with the average student earning 1 point higher on the oral report (see the Supporting Information for statistical comparison).

### Comparison between Virtual and In-Person Report Grade Outcomes

This assessment was designed to be completed in-person to be analogous to research collaboration conversations, but the COVID-19 pandemic shifted the way people communicate, and virtual meetings became the norm. Likewise, in an educational setting, in-person meetings shifted to virtual, even as our campus retained an in-person laboratory experience. This allowed a comparison between virtual oral lab reports in one semester (23 students) and in-person assessment the following semester (29 students). In these two environments, the same script and rubric were followed, along with instructor follow-up questions to clarify understanding. The scores across these two venues were nearly identical (virtual mean = 9.0 [0.8]; in-person mean = 9.0 [0.8]) and with no statistical difference (*t*(50) = 0.06, *p* = 0.96, *d* = 0.01).

### Different Instructors and Grade Outcomes

An important question when it comes to scalability and transferability of this work is whether the instructor matters, as most institutions employ teaching assistants or multiple instructors in their assessment of student work. While the same instructor taught all students in the lecture portion of the course, social distancing due to the pandemic required an additional lab section taught by a different instructor. Several steps were taken to minimize grading differences between instructors. First, instructors discussed the common script and rubric. Second, a virtual oral lab report conducted by the primary instructor was recorded with student permission and shared with the secondary instructor. This provided an example of follow-up questions that might be used to guide students to a better understanding during the assessment. While the numbers may be too small to make an accurate comparison (Instructor A with 44 students; Instructor B with 8 students), we looked at differences in scores in this unique situation, taking into account student performance on the standardized year-long organic chemistry exam from ACS, which serves as a final exam in the course. We found significant differences in oral report scores (Instructor A mean = 8.9 [0.8], Instructor B mean = 9.5 [0.4]; *t*(50) = 2.1, *p* = 0.038, *d* = 0.8) and scores on the final exam (Instructor A mean = 74.4 [18.5], Instructor B mean = 90.1 [11.3], *t*(50) = 2.3, *p* = 0.025, *d* = 0.9), but not in the average grades for the written reports (Instructor A mean = 8.0 [1.0], Instructor B mean = 8.0 [0.9], *t*(50) = 0.02, *p* = 0.98, *d* < 0.01). In a linear regression using instructor (coded as 0 = A, 1 = B), oral lab grade, and average written lab grade as predictors of final exam grades, both the oral exam grade (β = 0.31, p = 0.025) and average written lab grade (β = 0.36, p = 0.008) were significant predictors, while lab instructor (β = 0.22, p = 0.065) was not. Together, these results suggest that the differences seen between these two lab sections represent sampling error, and that better performance on both the oral and written lab reports was related to better performance on the final exam, independent of the lab instructor.

Overall, performance on the oral lab report was positively correlated with average grade for the written lab report (r(50) = 0.52, *p* < 0.001), as we might expect: students who perform well in a class often perform well across the range of course assessments. Interestingly, the linear regression found that performance on the oral lab report predicted final exam grades, even after controlling for the average written lab grade. The most likely reason for this may be that for each student, the three lab reports assessed different topics within the course. For individual students, the oral lab report grade reflected not only their overall performance in the course, but also their mastery of the specific concepts involved in that experiment, beyond what is captured by the average written lab grade.

## Discussion

Although this oral assessment was a single intervention with a small sample size, the results suggest students scored better under these assessment conditions, oral report scores predicted performance on the final exam independent of written report scores, and students described pedagogical outcomes consistent with previous studies in an open-ended survey response (Supporting Information), including an overwhelmingly positive experience that highlights communication, deep learning,^10–13^ and helpful feedback.^33–35^ Students generally scored higher on their oral report than on their written reports for other experiments, but this was not the case for every individual. Students often complete their written lab reports outside of lab hours and work with peers and online resources while writing, sometimes relying upon them heavily. In an oral assessment, students may collaborate during and after the experiment, but the instructor’s follow-up questions can assess an individual’s deeper understanding and clarify knowledge in real-time better than written assessment.

Virtual lab reports and an additional instructor in response to the pandemic allowed comparisons that are promising when thinking about implementation in other institutions or courses. The ability to conduct interviews in-person or virtually gives flexibility to instructors and teaching assistants who may need to work off-campus, during nonstandard hours, or without suitable office space for in-person interviews. Although the numbers that compare instructor scores are small, it is important to consider whether this assessment could be carried out by different individuals, as most institutions have a greater number of sections offered and multiple instructors leading them. Our results suggest there may be hope for reliable instructor grading.

### Instructor-Identified Benefits

Along with the improvement in student scores and student-reported positive experiences, both instructors shared observations while conducting the oral lab report. They noted that the pressure of the new assessment environment often motivated students to put more effort into understanding and internalizing their experimental results, which is consistent with student feedback. They did not observe student anxiety related to expectations for the assessment, which may be a benefit of using the familiar SWH-based format from written reports.

While scheduling may be a barrier to implementation of oral assessments, in this case, both instructors found these appointments far more enjoyable than the frustration of reading misguided student answers without being able to correct them efficiently. It regularly takes more than 15 min to grade a single written lab report and provide thoughtful feedback, so the grading time simply shifts. Completing assessments within a week of the experiment keeps everyone to task, there are no late lab reports from students, and the feedback is immediate. Further reflections can be found in the Supporting Information.

### Limitations

This study took place at a men’s college, but other reports have not found a gender difference in oral assessment performance.^25^ Smaller class sizes made implementation more straightforward, but it does limit this study to relatively small sample sizes. The oral and written lab report rubrics are not identical in the emphasis of all aspects of student understanding, which limits the direct comparison of student scores. However, we have shown that oral report scores are not significantly worse, which would be concerning for their implementation. There are promising results suggesting this work could scale to larger-enrollment courses. One concern that appears in the literature is instructor bias^24,25^ and, although the comparison in student scores shown here suffers from small numbers, there is some statistical evidence that instructor identity is not a predictor of student success. Further efforts to create consistent grading could include a training period with double-grading of oral reports to ensure a common understanding of the rubric and instructor expectations. If this work is to scale to larger programs, instructors may have to think creatively about how many oral assessments are feasible in the week following an experiment and we provide an example of spreading them out over three experiments. If teaching assistants are used, a more targeted training that addresses appropriate ways to follow up student errors with helpful leading questions could be useful, both for this assessment and for their overall development as teachers.

## Conclusions

While oral assessments are infrequently used in modern chemistry courses, the body of evidence to support their value continues to grow. The oral lab report described here provides an authentic opportunity for students to develop technical oral communication in a conversational assessment. Students can demonstrate their understanding and learn more during the conversation. The benefits of this format are not limited to a single experiment and can be experienced in virtual or in-person environments, suggesting that there are many opportunities to grow, adapt, and develop oral assessments, complementing the work that has been done to support writing in the sciences. Our hope is that this will lead to well-rounded students who are better prepared for real-world collaborative research.

## Supplementary Material





## Notes

Notes for Instructors and Grading Rubrics (PDF, DOCX)
