# Supplementary material for: Oral Reports in an Organic Chemistry Laboratory Curriculum
Source: J Chem Educ. 2026 Jun 23;103(7):3961–5. doi: 10.1021/acs.jchemed.5c00889 (PMC13374101; doi:10.1021/acs.jchemed.5c00889)
Supplement: Supplementary file 1 [file ed5c00889_si_001.pdf]

Supporting Information

Oral Reports in an Organic Chemistry Laboratory Curriculum

Sara A. Mehltretter[1], Neil Schmitzer-Torbert[2], Laura M. Wysocki[3]*

[1]Department of Rhetoric, Wabash College, Crawfordsville, IN 47933, USA

[2]Department of Psychology, Wabash College, Crawfordsville, IN 47933, USA

[5]Department of Chemistry, Wabash College, Crawfordsville, IN 47933, USA

*To whom correspondence should be addressed: wysockil@wabash.edu

Table of Contents

Notes for Instructors	S2–S4
Grading Rubrics	S5–S9
Written and Oral Prompt Comparison	S10-S11
Rubric Comparison for Skills Demonstrated	S12-S13
Statistical Comparison of Oral and Written Report Grade Outcomes	S14-S15
Open-Ended Student Survey Responses	S16
Instructor Reflections on Oral Lab Report	S17-S18

Instructor Notes for Conducting an Oral Lab Report

Oral Lab Reports last 10-15 minutes and incorporate many of the questions posed in the lab manual. In Organic Chemistry II, there are three labs that are worth 10 points and require a similar level of data analysis, so students can sign up for any of the three labs. The oral report is to be scheduled within a week after the lab itself. Students can use their lab manual as reference and should bring any data collected.

To begin each session, explain to the student that the questions I ask will sound a lot like a written report, but that the oral format allows for more of a discussion. The student doesn't have to have the right answer immediately, but rather it is ok to talk through the explanation to get to a conclusion.

Lab 1: Wittig Reaction and Complex NMR

Questions asked during the oral lab report interview:

1. What are the Beginning Questions for the lab?
2. What is a safety concern that you had to keep in mind during the experiment? (for this lab, NMR safety is important)
3. If a classmate asked you what he needed to remember about the procedure for the lab, what would you tell him? (The student needs to remember something specific about the experimental procedure, including how much propionaldehyde they ended up adding.)
4. What are some observations that you made during the experiment?
5. Draw the reaction on the board so that we can refer to it for the following questions.
6. Draw the zwitterion form of the ylide. Where in this reaction is the nucleophile? Where is the electrophile? Why would this be called a "stabilized" Wittig reaction?
7. Let's look at the NMR data for your product, specifically the peaks at δ7.0 and 5.8 ppm. For each peak, name the coupling pattern that you see. Which proton in the product does each peak represent? Explain.
8. Is your product the *E* or *Z* alkene? What is your evidence for that claim? Is your evidence convincing in this case?
9. What are your reflections for this lab? This should not be a summary. Was there anything surprising or unexpected based on what we learned in class? Are there any further experiments you would like to do?

Lab 2: Acetal Formation

Questions asked during the oral lab report interview:

1. What are the Beginning Questions for the lab?
2. What is a safety concern that you had to keep in mind during the experiment?
3. If a classmate asked you what he needed to remember about the procedure for the lab, what would you tell him? (the student needs to remember something specific about the experimental procedure – for this experiment, temperature is important)
4. What are some observations that you made during the experiment? (for this experiment, the appearance of the reaction over the course of the hour is important)
5. Draw the reaction on the board so that we can refer to it for the following questions.
6. Why would water typically be a bad solvent for a reaction like this (Le Chatelier)? Why does it work here? (student should refer to appearance of precipitate and solubility of product)
7. Keeping in mind the answer to the previous question, why does this reaction stop at the monoacetal even though there is an excess of benzaldehyde? How would you prepare the diacetal?
8. Let's look at your data. Analyze the main peaks in your IR and look at your melting point. What does this tell you about the purity of the product?
9. Bring out the molecular model of the product (I usually have one made) and set it on the table. Analyze the NMR using shift, integration, and splitting and tell me which protons are represented in each peak. How many types of protons are on the model? (In this conversation, students often place equatorial and axial protons on the same carbon in one "group" – this leads to the question of whether they are homotopic, enantiotopic, or diasterotopic and what that means for NMR analysis).
10. Do you think you made the mono-acetal product? What is your evidence for that claim? Is your evidence convincing in this case?
11. What are your reflections for this lab? This should not be a summary. Was there anything surprising or unexpected based on what we learned in class? Are there any further experiments you would like to do?

Lab 3: Combinatorial Synthesis of Esters

Questions asked during the oral lab report interview:

1. What are the Beginning Questions for the lab?
2. What is a safety concern that you had to keep in mind during the experiment?
3. If a classmate asked you what he needed to remember about the procedure for the lab, what would you tell him? (the student needs to remember something specific about the experimental procedure – for this lab, temperature of reaction is important)
4. What are some observations that you made during the experiment? (for this lab, the smell of methyl salicylate is important)
5. Draw the general reaction on the board so that we can refer to it for the following questions.
6. What is the role of the acid in this reaction? What are the nucleophilic/electrophilic sites? This reaction is in equilibrium. What can we do to drive this reaction forward (what did we do and what could we do better)?
7. This experiment suggested there is one and only one combination that smells like wintergreen. What is your claim?
8. Let's look at your data. Explain to me the evidence that led to your claim. How strong is that evidence? Is your evidence convincing in this case?
9. Your lab manual asks questions about a false positive, which is a test that inaccurately identifies activity. If you wanted to be sure of your identification, how many possible combinations would there be with a false positive? Explain.
10. Your lab manual asks questions about a false negative, which is a test that should identify activity, but does not. If you wanted to be sure of your identification, how many possible combinations would there be with a false negative? Explain.
11. Which one is more desirable – a false positive or false negative?
12. What are some limitations to this experimental setup? What are some benefits to the experimental setup?
13. What are your reflections for this lab? This should not be a summary. Was there anything surprising or unexpected based on what we learned in class? Are there any further experiments you would like to do?

Oral Lab Report Rubric

Skill	Points Possible	Points Earned
Frame the experiment and its goals (Beginning questions, Safety, Procedure, Write reaction)	1.5	
Make observations	0.5	
Theory behind experiment	1	
Data analysis	3	
Claim and use of evidence	1.5	
Reflect on experiment (connect to class, limitations/benefits, and further experiment)	1.5	
Ability to communicate ideas in a meaningful way	1	
Total	10	

The general rubric reported here describes the points available for a variety of skills that were assessed in the oral lab report. The point values for student performance of each skill should be specific to the experiment performed and agreed upon by all instructors involved in administering the assessment. For example, the category of "Claim and use of evidence" might distribute the 1.5 points into 0.5 points for the accuracy of the claim, 0.5 points for the breadth of the claim (i.e. does it answer key questions for all parts of the experiment), and 0.5 points for the use of evidence (i.e. does it include all the data collected). The

category of “Ability to communicate ideas in a meaningful way” might award full points to students who use technical language accurately and clearly explain their thought process. Point deduction may occur if students use jargon inappropriately or if they use the correct term but cannot explain its application. On the other hand, instructors may agree to award full points to students who can explain a concept clearly in their own words without using the technical terms. Best practices include designing some flexibility in rubric definition.[S1]

[S1] Gehrke, P. Creating more equitable rubrics to reduce discrimination and inequities in public-speaking courses. *Communication Teacher* **2025**, *39* (2), 163–179. DOI: 10.1080/17404622.2024.2372345

Wittig Written Lab Report Rubric

Skill	Points Possible	Points Earned
Frame the experiment and its goals (Beginning questions, Safety, Procedure, Write reaction)	3	
Table of Reagents	1	
Make observations	1	
Theory behind experiment	1	
Claim	1	
Evidence and Data Analysis	2.5	
Reflect on experiment (connect to class, limitations/benefits, and further experiment)	0.5	
Total	10	

Acetal Formation Written Lab Report Rubric

Skill	Points Possible	Points Earned
Frame the experiment and its goals (Beginning questions, Safety, Procedure, Write reaction)	2	
Table of Reagents	0.5	
Make observations	1	
Percent Yield Calculation	1	
Theory behind experiment	2	
Claim	0.5	
Evidence and Data Analysis	2	
Reflect on experiment (connect to class, limitations/benefits, and further experiment)	1	
Total	10	

Fischer Esterification Written Lab Report Rubric

Skill	Points Possible	Points Earned
Frame the experiment and its goals (Beginning questions, Safety, Procedure, Write reaction)	3.5	
Table of Reagents	0.5	
Make observations	1	
Theory behind experiment	2	
Claim	1	
Evidence and Data Analysis	1	
Reflect on experiment (connect to class, limitations/benefits, and further experiment)	1	
Total	10	

Written and Oral Report Prompt Comparison

Comparison between selected language from written lab report instructions and oral lab report prompt for one of the experiments described:

**Written report instructions**	**Oral report prompt (ex: Wittig)**
Beginning Questions<br><br>… write a question or two that can be answered by doing the experiment. Talk about what analytical techniques you will use to answer these questions.<br><br>Balanced equations for any ***reactions*** to be carried out should be included.	1. What are the Beginning Questions for the lab?<br><br>5. Draw the reaction on the board so that we can refer to it for the following questions.
Safety<br><br>Any particularly hazardous operations or substances should be noted.	2. What is a safety concern that you had to keep in mind during the experiment?
Tests/Procedures<br><br>The experimental procedure should be an outline in your own words.	3. If a classmate asked you what he needed to remember about the procedure for the lab, what would you tell him? (The student needs to remember something specific about the experimental procedure, including how much propionaldehyde they ended up adding.)
Table of Reagents<br><br>Information that could be pertinent to an experiment includes: molecular weight of all compounds, melting point of solids, boiling point and density of liquids, and concentration of all solutions.	No corresponding question
Data/Observations<br><br>Make sure you **label** and record all the data you collect, with appropriate significant figures for measurements.	4. What are some observations that you made during the experiment?
Claim<br><br>The claim should be a one- or two-sentence statement about the results of your laboratory work that might answer the beginning questions.	8. Is your product the *E* or *Z* alkene?<br>(Note: this is the key question for this experiment, which is stated in the lab manual.)

Evidence<br><br>This is a written explanation that supports your claims and utilizes the data, observations, and analysis that you have performed during the experiment. Explain the meaning behind data and calculations. Spectra and chromatograms need to be interpreted in order to count as evidence – simply referring to them is not enough. How strong is your evidence in supporting your claim? ***Analyze*** and ***Explain*** your results.	7. Let's look at the NMR data for your product, specifically the peaks at δ7.0 and 5.8 ppm. For each peak, name the coupling pattern that you see. Which proton in the product does each peak represent? Explain.<br><br>8. ... What is your evidence for that claim? Is your evidence convincing in this case?
Reflection<br><br>Refer back to your initial question. Have your ideas changed? How strong is the claim you made? If it is not strongly supported, why? Do you have a new question? How do your results compare to what you would expect based on literature values and what you have learned in lecture?	9. What are your reflections for this lab? This should not be a summary. Was there anything surprising or unexpected based on what we learned in class? Are there any further experiments you would like to do?
(Note: some labs have additional questions stated in the lab manual. This is specific to the Wittig experiment.)<br><br>Draw the zwitterion resonance structure of (carbethoxymethylene)triphenylphosphorane. Identify the nucleophilic site and the electrophilic site in the reaction performed in this week's lab.	6. Draw the zwitterion form of the ylide. Where in this reaction is the nucleophile? Where is the electrophile? Why would this be called a "stabilized" Wittig reaction?

Point Distribution According to Skill Demonstrated for Oral and Written Lab Report Rubrics for Acetal Formation Experiment

Questions asked during Acetal Formation Oral Lab Report	Skill	Points Possible In Oral Report	Points Possible in Written Report
1. What are the Beginning Questions for the lab?<br>2. What is a safety concern that you had to keep in mind during the experiment?<br>3. If a classmate asked you what he needed to remember about the procedure for the lab, what would you tell him? (The student needs to remember something specific about the experimental procedure, including how much propionaldehyde they ended up adding.)<br>5. Draw the reaction on the board so that we can refer to it for the following questions.	Frame the experiment and its goals (Beginning questions, Safety, Procedure, Write reaction)	1.5	2
	Complete appropriate Table of Reagents	N/A	0.5
4. What are some observations that you made during the experiment? (for this experiment, the appearance of the reaction over the course of the hour is important)	Make observations	0.5	1
6. Why would water typically be a bad solvent for a reaction like this (Le Chatelier)? Why does it work here? (student should refer to appearance of precipitate and solubility of product)<br>7. Keeping in mind the answer to the previous question, why does this reaction stop at the monoacetal even though there is an excess of benzaldehyde? How would you prepare the diacetal?	Theory behind experiment	1	2
8. Let's look at your data. Analyze the main peaks in your IR and look at your melting point. What does this tell you about the purity of the product?<br>9. Bring out the molecular model of the product and set it on the table. Analyze the NMR using shift, integration, and splitting and tell me which protons are represented in each peak. How many types of protons are on the model?	Data analysis	3	2.5
10. Do you think you made the mono-acetal product? What is your evidence for that claim? Is your evidence convincing in this case?	Claim and use of evidence	1.5	1
11. What are your reflections for this lab? This should not be a summary. Was there anything surprising or unexpected based on what we learned in class? Are there any further experiments you would like to do?	Reflect on experiment (connect to class, limitations/benefits, and further experiment)	1.5	1

	Ability to communicate ideas in a meaningful way	1	N/A
	Total	10	10

Statistical Comparison of Oral and Written Report Grade Outcomes

A total of 52 students completed these assessments in two offerings of the Organic Chemistry II lab (23 in spring 2021 and 29 in spring 2022). Students scored better on oral lab reports compared to written lab reports for the same experiment (Figure S1). This difference is most apparent in the Wittig experiment, with the mean oral lab report score of 9.2 [SD = 0.8] out of 10 points and the mean written lab report score of 7.8 [1.1] ($t(50) = 4.7$, $p < 0.0001$, Cohen's $d = 1.3$). The difference in scores is also favorable for the acetal experiment, with the oral lab report mean at 8.5 [0.8] compared to 7.7 [1.3] in the written lab report ($t(50) = 2.6$, $p = 0.01$, $d = 0.7$). For the esterification experiment, the oral lab report mean was 9.2 [0.6] compared to the written lab report mean of 8.4 [0.8] ($t(50) = 3.4$, $p = 0.001$, $d = 1.0$).

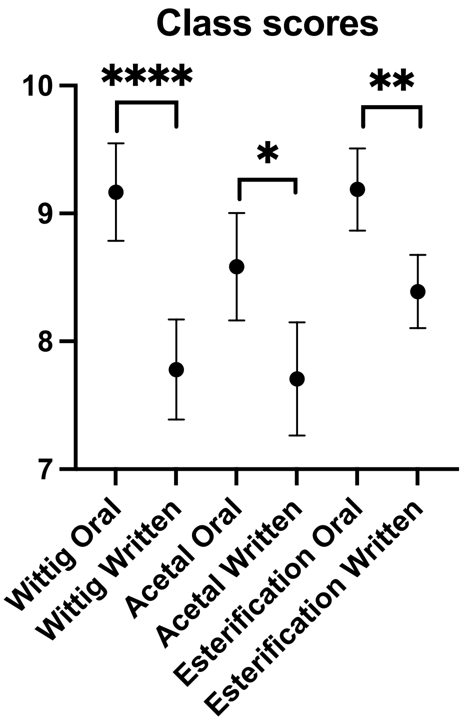

Figure S1. Comparison of class scores for oral lab reports and written lab reports for three experiments: Wittig (oral $N = 18$, written $N = 34$), Acetal (oral $N = 18$, written $N = 34$), and Esterification (oral $N = 16$, written $N = 36$). *p = 0.01, **p = 0.001, ****p < 0.0001

Students generally scored better on the oral lab report than they did on the average of their two written lab reports (Figure S2). The mean score for the oral lab report was 9.0 [0.8], while the mean score for written lab reports was 8.0 [0.9] ($t(51) = 8.4$, $p < 0.0001$, $d = 1.2$). Across the

sample, forty-five students saw a higher score on the oral lab report, four had equal scores for both report formats, and three had a higher average for their written lab reports.

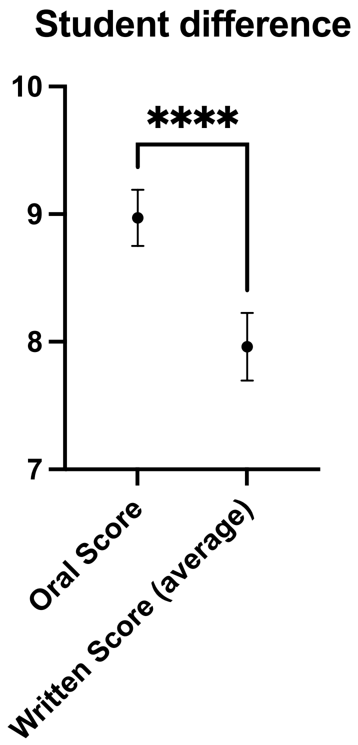

Figure S2. Comparison of student scores for oral lab reports and the average of their written lab reports for the other two experiments in this study. ****$p < 0.0001$

Open-Ended Student Survey Responses

Positive learning outcomes typically associated with oral assessments were apparent in student survey responses at the end of the semester. One of the questions asked students to "please add any comments about the oral lab report experience." The open-ended responses from this question were valence coded and inductively thematically coded (by L.M.W. and S.A.M.), with differences reconciled through researcher discussion.[S2] On valence, student responses were overwhelmingly positive. Thematic responses aligned into several themes that are consistent with previous reports from the literature associated with oral assessment. Out of 51 students responding, 46 expressed a positive comment about oral lab reports, 3 were neutral, 1 was mixed, and 1 was negative, expressing that they felt ill-prepared and prefer the written format. The importance of developing communication skills came up in 20 of the responses and, along the same lines, 9 students appreciated the change in format mid-semester to break up the monotony of assessment format. In agreement with previous reports that emphasize student learning,[10-13] 17 students talked about making stronger connections to the material in the oral format or developing a greater understanding of the topic covered by the experiment. Relatedly, 4 of the students volunteered that they underwent greater preparation for the oral lab report than they typically would for a written lab report and 2 felt that they should have done more preparation for this format. The immediate feedback available during the conversation in this format was mentioned as a positive aspect by 11 students, while one student asked for more feedback after the discussion ended. Interestingly, students reported a variety of feelings about the assessment, with 5 students reporting positive feelings such as "conversational" or "laid-back," with another 6 students pointing out feelings of uncertainty and/or stress related to their experience.

[S2] Asen, R.; Gurke, D.; Conners, P.; Solomon, R.; Gumm, E. Research Evidence and School Board Deliberations: Lessons from Three Wisconsin School Districts. *Sage* **2013**, *27* (1), 33–63. DOI: 10.1177/0895904811429291

Instructor Reflections on Oral Lab Report

The students self-identified deeper understanding as a benefit of the oral lab report, which was clear to the instructors as well. Overall, students were able to communicate technical information about experiments in an informal conversation, similar to the instructors' experiences in professional collaborative research situations.

When a student answered a question incorrectly, both instructors followed up with further questions to probe student understanding. Sometimes, students could self-correct when given the opportunity to think about a question stated differently. Even when this wasn't the case and the instructor had to reveal an answer, the student had the opportunity to better understand the concept and apply what they just learned to subsequent discussion of the experiment and its data. This opportunity, which is only available with immediate feedback, led to "aha" moments for students in real-time as they analyzed data and strengthened their confidence. Under the conditions of a written lab report, a conceptual misunderstanding early in the report can lead to confusion and mistakes later in the report, which is only revealed in delayed written feedback that may or may not be seen, understood, and internalized by the student.[4,24] Even when students see and understand the written feedback, in our experience, they rarely follow up to have a conversation about how to rectify their misunderstanding. This aspect of the oral lab reports was incredibly satisfying as an instructor and students frequently commented that they understood the experiment better at the end of the oral lab report than they did coming into the meeting, which is consistent with feedback conversations.[27,28]

An unanticipated benefit of the immediate feedback given in the oral lab report is that it can serve to guide students in instructor expectations for their work. While the students participating in this course had several written reports using the SWH-inspired format, some still weren't meeting expectations for different sections of the report, perhaps because the delayed written feedback wasn't informative enough or they were uncomfortable asking for further help. The oral lab report provided a natural opportunity to discuss the critical information required when writing data analysis, the key parts to include in beginning

questions, or what to consider when describing a further experiment. This valuable opportunity provides the instructor with a window into student confusion and it provides the students with a window into instructor grading. Future research may include evaluating student performance on various aspects of subsequent written reports.
